# Maternal periconceptional environmental exposure and offspring with congenital heart disease: a case–control study in Guangzhou, China

**DOI:** 10.1186/s12884-023-05355-5

**Published:** 2023-01-24

**Authors:** Di Xiao, Weidong Li, Wei-Hong Zhang, Zihao Wen, Weijian Mo, Ciyong Lu, Lan Guo, Li Yang

**Affiliations:** 1 Department of Comprehensive Maternal and Child Health, Guangzhou Women and Children’s Medical Center, Guangdong Provincial Clinical Research Center for Child Health, Guangzhou, 510623 China; 2 International Centre for Reproductive Health (ICRH), Department of Public Health and Primary Care, Ghent University, Ghent, Belgium; 3 School of Public Health, Université libre de Bruxelles (ULB), 1070 Bruxelles, Belgium; 4 Department of Medical Statistics and Epidemiology, School of Public Health, Sun Yat-sen University, Guangzhou, Guangdong China

**Keywords:** Congenital heart defects, Living near main roads, Family relationships, Incense burning, Housing renovation

## Abstract

**Background:**

Congenital heart defects (CHDs) are a major global health problem, yet their crucial environmental risk factors are still unclear. We aimed to explore the associations between maternal periconceptional environmental exposures and all CHDs, isolated and multiple CHDs and CHDs subtypes.

**Method:**

A case–control study including 675 infants with CHDs and 1545 healthy controls was conducted. Participating mothers who delivered in Guangzhou from October 2019 to November 2021 were recruited. To examine the independent associations between maternal periconceptional environmental exposure and offspring with CHDs, we calculated odds ratios (ORs) and 95% confidence intervals (CIs) using multivariable logistic regression model.

**Results:**

Maternal exposure to living near main roads [adjusted OR (aOR) = 1.94, 95% CI = 1.06–3.56] and housing renovation (aOR = 1.94, 95% CI = 1.03–3.67) during the periconceptional period were positively related to a greater risk of all CHDs, similar results were also found in isolated CHDs rather than multiple CHDs. Additionally, living near main roads was positively associated with secundum atrial septal defect/patent foramen ovale (aOR = 2.65, 95% CI = 1.03–6.81) and housing renovation was strongly positively associated with ventricular septal defect (aOR = 5.08, 95% CI = 2.05–12.60). However, no association was observed between incense burning and family relationships and all CHDs, isolated and multiple CHDs and CHDs subtypes.

**Conclusion:**

Living near main roads and housing renovation during the periconceptional period are significantly associated with the increased risks for all CHDs and isolated CHDs. Further study is needed to extend sample size to explore the effects of time and frequency of burning incense and family relationships on CHDs in offspring.

**Supplementary Information:**

The online version contains supplementary material available at 10.1186/s12884-023-05355-5.

## Introduction

Congenital heart defects (CHDs) constitute the most prevalent congenital anomalies [1], affecting millions of newborns annually [2]. It is estimated that the global birth prevalence of CHDs is 8 to 12 infants per 1000 births [3]. In the United States, CHDs affect approximately 1% of births [4]. In Sweden, the prevalence of CHDs increased steadily from 5.7 to 20 per 1000 live births from 1970 to 2017 [5]. In Western Australia, approximately 11.5‰ of liveborn children were born with CHDs from 1990 to 2016 [6]. In China, according to a large prospective multicenter screening study, the prevalence of CHDs was 8.98‰ [7]. Since CHDs greatly influence children’s quality of life and cause a serious financial burden of families and society as a whole [8], posing a significant global threat to public health [9].

The cause of CHDs is multifactorial [10] and largely unknown [11]. Both environmental and genetic factors contribute to the development of CHDs [11]. Increasing epidemiological evidence have highlighted the importance of the environment in CHDs, with up to 30% of cases being explained by environmental factors [3], such as maternal factors involving maternal diabetes mellitus, obesity, maternal smoking, dietary folate intake, alcohol consumption, certain drug use during pregnancy, exposure to air pollutants and life events, as well as paternal factors (e.g., advanced age) [12–15]. As CHDs represent a significant public health issue, understanding the causes of CHDs, especially those factors that can be prevented, is crucial for their primary prevention [14].

Household incense burning is a common practice in the Asia-Pacific region [16], and is used for ritual or religious purposes [17]. A cohort study involving 10,563 pregnant women from Guangzhou, China showed that 25.4% of women reported that incense was burnt in their household during early pregnancy [18]. As an important source of indoor air pollution, incense burning produces smoke (fumes) containing numerous hazardous air pollutants [18]. Recently, a study found that maternal incense burning exposure was linked to a higher risk of adverse birth outcomes, such as low birth weight and small for head circumference [19]. Although evidence indicates an association between maternal exposure to incense burning and adverse birth outcomes, little attention has been paid to investigate the potential effect of this exposure on CHDs.

Living near traffic is a multifaceted exposure representing heightened exposure to numerous hazardous air pollutants [e.g., fine particulate matter (PM2.5), nitrogen oxides, heavy metals, ultrafine particles], noise, and other factors, and has been related to elevated risks of lower fecundability [20], preeclampsia [21], and lung cancer [22]. Emerging evidence suggests that a contradictory association between residential proximity to major roads and adverse birth outcomes (preterm birth) [23, 24]. However, limited studies thus far have examined the influence of living near main roads on CHDs in offspring [25]. As hundreds of millions of people live near to major roads around the world [26], we aim to address the potential association between living near main roads and CHDs in offspring, which might provide novel insights into understanding the mechanisms of CHDs.

Additionally, evidence has also shown that acute and chronic stress increase the risk of a variety of adverse pregnancy outcomes, such as low birthweight, preeclampsia, spontaneous preterm birth (PTB), and neonatal morbidity [27–29]. Recently, Gu et al. [30] reported that maternal stress and stressful life events during pregnancy increased the risk of offspring CHDs. As a stressor, family conflict is a relationship challenge, and little is known about the association between poor relationships with family and CHDs in offspring.

Housing renovations, as a type of indoor air pollution, have been identified as a public health threat, particularly for fetuses and children [1]. Moreover, a previous study suggested that housing renovations during the periconceptional period may be associated with CHDs in offspring [1].

Evidence has demonstrated that the periconceptional period is a critical window of exposure that can influence the growth and development of offspring [31]. The identification of potentially modifiable risk factors in a critical window for CHDs provides opportunities for public health strategies for CHD prevention to improve birth outcomes [18]. Therefore, the present research aimed to explore the associations between maternal periconceptional environmental exposure (family relationships, housing renovation, living near to major roads, and incense burning) and all CHDs, isolated and multiple CHDs as well as isolated CHDs subtypes.

## Materials and methods

### Data and participants

The present study was a case–control study, which data collection was performed in Guangzhou, Guangdong Province, China. In this study, participating mothers who delivered in Guangzhou from October 2019 to November 2021. They were recruited by 126 community health service centers that provided them with maternal health services during their pregnancy through 42 days postpartum. Participating mothers of cases and controls completed a questionnaire administered over the telephone by trained doctors in each community health service center.

CHDs Cases were involved met the following criteria: (a) singleton pregnancy (b) delivered from October 2019 to November 2021; (c) gestational age ≥ 28 weeks; and (d) perinatal children (including single live births and stillbirths) with diseases coded as Q20-Q26 in the International Classification of Diseases, 10th Revision, Clinical Modification (ICD-10-CM) diagnosed with at least one kind of CHD without any other birth defects according to the guidelines of the Maternal and Child Health Monitoring Manual in China [32]. The CHD cases in this study included live births and stillbirths. All CHD cases were diagnosed from 28 weeks after pregnancy to 7 days by pediatric cardiologists after clinical diagnosis was performed by heart auscultation and fetal and neonatal echocardiography according to ICD-10 classification criteria. If necessary, a CHD case was further diagnosed by computed tomography, cardiac catheterization, surgery, or autopsy. “Isolated CHD” (only one type of cardiac malformation) and “Multiple CHDs” (more than one cardiac malformation) were included [33]. The details of types of 675 CHD cases were showed in Supplementary Table 1. The controls were the healthy newborn infants without any birth defects.

The exclusion criteria were as follows: (a) twin and multiple births; (b) gestational age < 28 weeks; (c) delivery outside the range of October 2019 to November 2021; and (d) infants with birth defects other than CHDs; (e) infants with at least one kind of CHDs and other birth defects.

All CHD cases and controls were double checked via the Guangzhou Maternal and Children Health Care Information System (MCHCIS). Guangzhou MCHCIS was officially established to collect information on pregnant females and their offspring from all community health service centers and midwifery agencies in Guangzhou. All cases of birth defects are reported to the government administration via the Guangzhou MCHCIS, which provided data quality assurance.

### Ethics

This study was approved by the Guangzhou Women and Children’s Medical Center Institutional Review Board (No. 201934001). Then, after the study procedures had been fully described, informed consent was obtained from all individual participants included in the study.

### Maternal exposure measurement and definitions

In this study, the periconceptional period (3 months before pregnancy through the first trimester) [34] was defined as the exposure window. Family relationships, incense burning exposure, housing renovation, and living near main roads during the periconceptional period were evaluated.

Family relationships during the periconceptional period were assessed according to the participating mothers’ self-rating of their relationships with their family members (categorized into “average or above-average = 1”, “below the average = 2”).

Living near main roads was measured by the questionnaire item of “Was your bedroom less than 50 meters away from the main road during the periconceptional period?”, with responses coded as “no = 0” and “yes” = 1.

Maternal exposure to housing renovation was assessed via the following question “From 3 months before pregnancy through the first trimester, did your household undertake house renovation or interior finishing?” with responses coded as “no = 0” and “yes” = 1.

Incense burning exposure was measured by asking participating mothers the following questionnaire item: “During 3 months before pregnancy through the first trimester, did your household have burning incense?” The response options were (1) “never”, and (2) “yes”. Participants who reported “(1)” were classified as “no exposure”. Responses were defined as exposure when the selected answers was “(2)”.

### Covariates

The demographic variables included maternal age at delivery, paternal age at delivery, family monthly income (< 5000, 5000–10,000,10,000–20,000 and ≥ 20,000 RMB), and maternal passive smoking. Maternal passive smoking was defined as living with someone who smokes at home or other places (e.g., workplaces, restaurants etc.) during pregnancy.

Obstetric variables, such as preterm birth (no/yes), birth weight (g), parity, gravidity, mode of conception (planned pregnancy, unplanned pregnancy or assisted reproduction), maternal folic acid use (no/yes), threatened abortion (hemorrhage in early pregnancy), and maternal reproductive history of birth defects (no/yes) were also investigated. Furthermore, maternal history of internal diseases (no/yes), maternal diabetes (no/yes), maternal history of CHD (no/yes), and maternal hyperthyroidism (no/yes) were assessed before pregnancy. “Taking medicine” was defined as taking any drugs such as antibiotics, antipyretics, antibiotics, anticancer drugs, or hormones during the first trimester.

### Statistical analysis

First, descriptive analyses were used to describe the demographic characteristics, obstetric variables and maternal periconceptional environmental exposure. Data are expressed as means and standard deviations for continuous variables or as frequencies and percentages for categorical variables. To compare participants with and without CHDs, chi-square tests were conducted for categorical variables, and t tests were conducted for continuous variables. Second, the odds ratios (ORs) and 95% confidence intervals (CIs) were calculated by applying univariable logistic regression models to test the relationships between maternal periconceptional environmental exposures and CHDs. Finally, after adjusting for potential confounders with a value of *P* < 0.05 in the univariate analyses and potentially relevant variables reported in the literature, the independent associations of maternal periconceptional environmental exposures with all CHDs, isolated and multiple CHDs were determined via multivariable logistic regression models. Then we further assessed the independent associations between maternal periconceptional environmental exposures and major subtypes of CHDs of this study, including patent ductus arteriosus (PDA), ventricular septal defect (VSD) and secundum atrial septal defect (ASD)/patent foramen ovale (PFO). SPSS software v. 23.0 (SPSS, Inc.) and R version 4.2.1 were used to conduct all statistical analyses.

Statistical significance was set at two-tailed *P* value less than 0.05.

## Results

### Characteristics of the study population

**Fig. 1:**
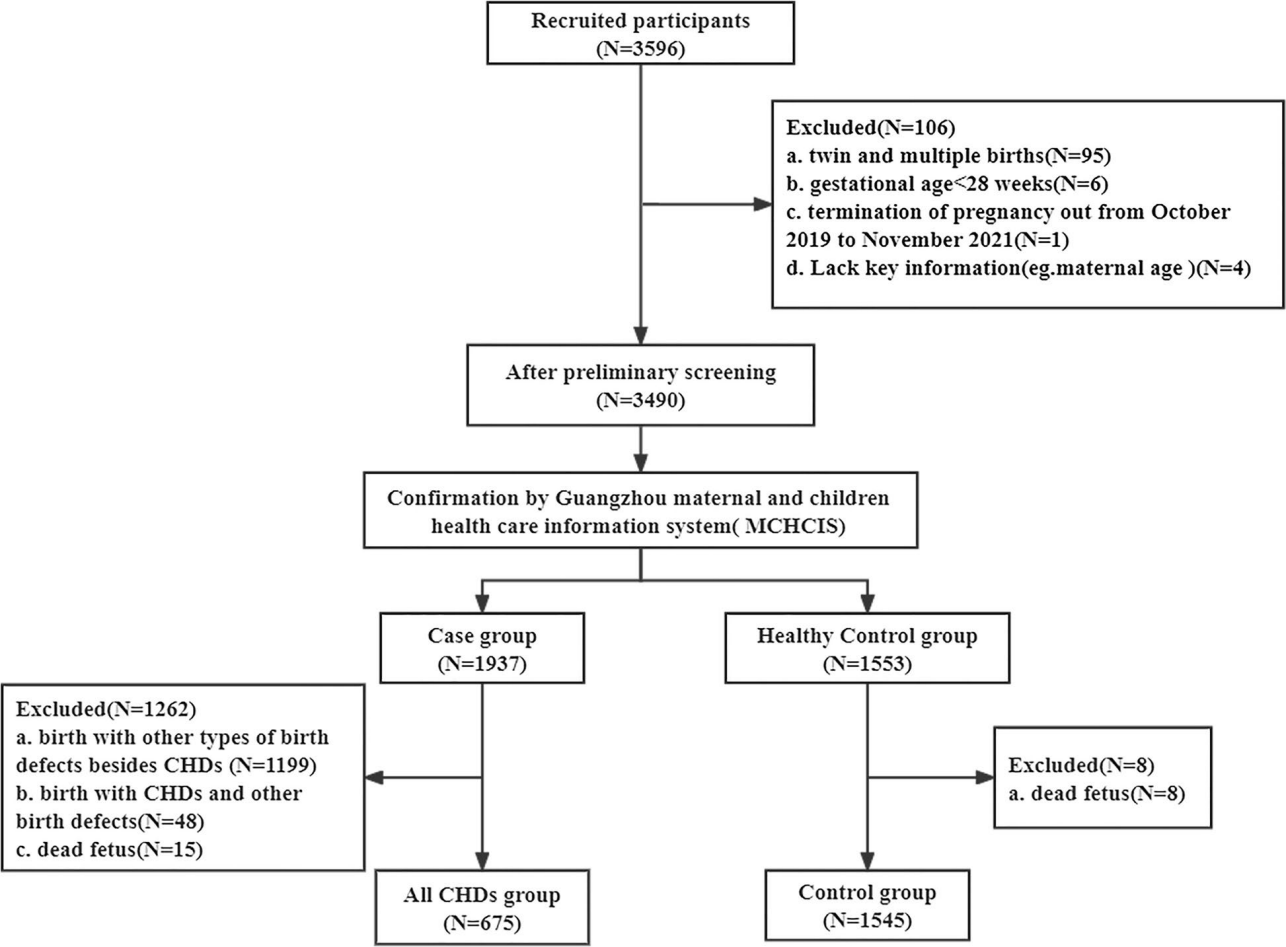
Flow chart of study participants

A total of 675 infants with CHDs and 1545 healthy controls were included in our study (Fig.1). Maternal characteristics for infants with and without CHDs are presented in Table 1. Compared with healthy controls, CHD cases were more likely to have lower parity, infant with preterm birth, maternal passive smoking during pregnancy, lack of maternal folic acid use, assisted reproduction, threatened abortion, as well as have a history of internal diseases, CHD, hyperthyroidism, and reproductive history of birth defects before pregnancy (all *P* < 0.05).

**Table 1:** Maternal characteristics among infants with and without congenital heart diseases (*n* = 2220)

Variables	Healthy Controls		All CHDs Cases		*P*
	*n* = 1545	%	*n* = 675	%	
Maternal age at delivery (year)					0.568
< 35	1308	84.7	565	83.7	
≥ 35	237	15.3	110	16.3	
Paternal age at delivery (Mean ± SD, year)	31.7 ± 5.1		32.2 ± 5.1		0.053
Child sex					0.078
Male	834	54.0	337	49.9	
Female	711	46.0	338	50.1	
Family monthly income (per month/RMB)					0.396
< 5000	50	3.2	27	4.0	
5000–10,000	467	30.3	216	32.1	
10,000–20,000	718	46.6	313	46.6	
≥ 20,000	306	19.9	116	17.3	
Gravidity (Mean ± SD)	2.09 ± 1.16		2.07 ± 1.18		0.650
Parity (Mean ± SD)	1.57 ± 0.61		1.51 ± 0.65		0.034*
Maternal passive smoking					0.005*
No	1476	95.5	625	92.6	
Yes	69	4.5	50	7.4	
Maternal use of folic acid					0.005*
No	60	3.9	45	6.7	
Yes	1478	96.1	629	93.3	
Mode of conception					< 0.001*
Planned pregnancy	1039	67.8	418	62.2	
Unplanned Pregnancy	475	31.0	231	34.4	
Assisted reproduction	19	1.2	23	3.4	
Threatened abortion					0.046*
No	1466	94.9	626	92.7	
Yes	79	5.1	49	7.3	
Birth weight (Mean ± SD, g)	3165.2 ± 432.5		3126.0 ± 578.0		0.114
Maternal history of internal diseases					0.043*
No	1537	99.5	666	98.7	
Yes	8	0.5	9	1.3	
Preterm birth					0.006*
No	113	7.3	73	10.8	
Yes	1432	92.7	602	89.2	
Maternal diabetes					0.941
No	1534	99.3	670	99.3	
Yes	11	0.7	5	0.7	
Maternal history of CHD					0.004*
No	1545	100	670	99.3	
Yes	0	0	5	0.7	
Maternal reproductive history of birth defects					< 0.001*
No	1542	99.8	659	97.6	
Yes	3	0.2	16	2.4	
Taking medicine					< 0.001*
No	1402	90.7	573	84.9	
Yes	143	9.3	102	15.1	
Maternal hyperthyroidism					0.048*
No	1542	99.8	670	99.3	
Yes	3	0.2	5	0.7	

*P < 0.05

Abbreviations: *CHD* congenital heart disease

### Characteristics of maternal environmental exposures

Table 2 highlights the comparison of maternal environmental exposures during the periconceptional period of characteristics between CHD cases and healthy control groups. Mothers of CHD cases reported a higher frequency of living near main roads and housing renovation compared with the controls (3.3% vs. 1.6, and 2.8% vs. 1.5%, respectively) (all *P* < 0.05). However, no significant difference was observed between the CHD cases and control groups in family relationships (*P* = 0.987) and incense burning (*P* = 0.260) during the periconceptional period.

**Table 2:** Association between maternal environmental exposures and offspring congenital heart disease

Variables	Healthy Controls		CHDs Cases		*P*
	n	%	n	%	
**Periconceptional maternal exposure**					
Family relationships					0.987
Average or above-average	1318	85.3	576	85.3	
Below the average	227	14.7	99	14.7	
Housing renovation					0.035*
No	1522	98.5	656	97.2	
Yes	23	1.5	19	2.8	
Living near main roads					0.009*
No	1521	98.4	653	96.7	
Yes	24	1.6	22	3.3	
Incense burning					
No	1376	89.1	590	87.4	0.260
Yes	169	10.9	85	12.6	

*P < 0.05

Abbreviations: *CHD* congenital heart disease

### Association of maternal periconceptional environmental exposure and offspring congenital heart disease

**Fig. 2:**
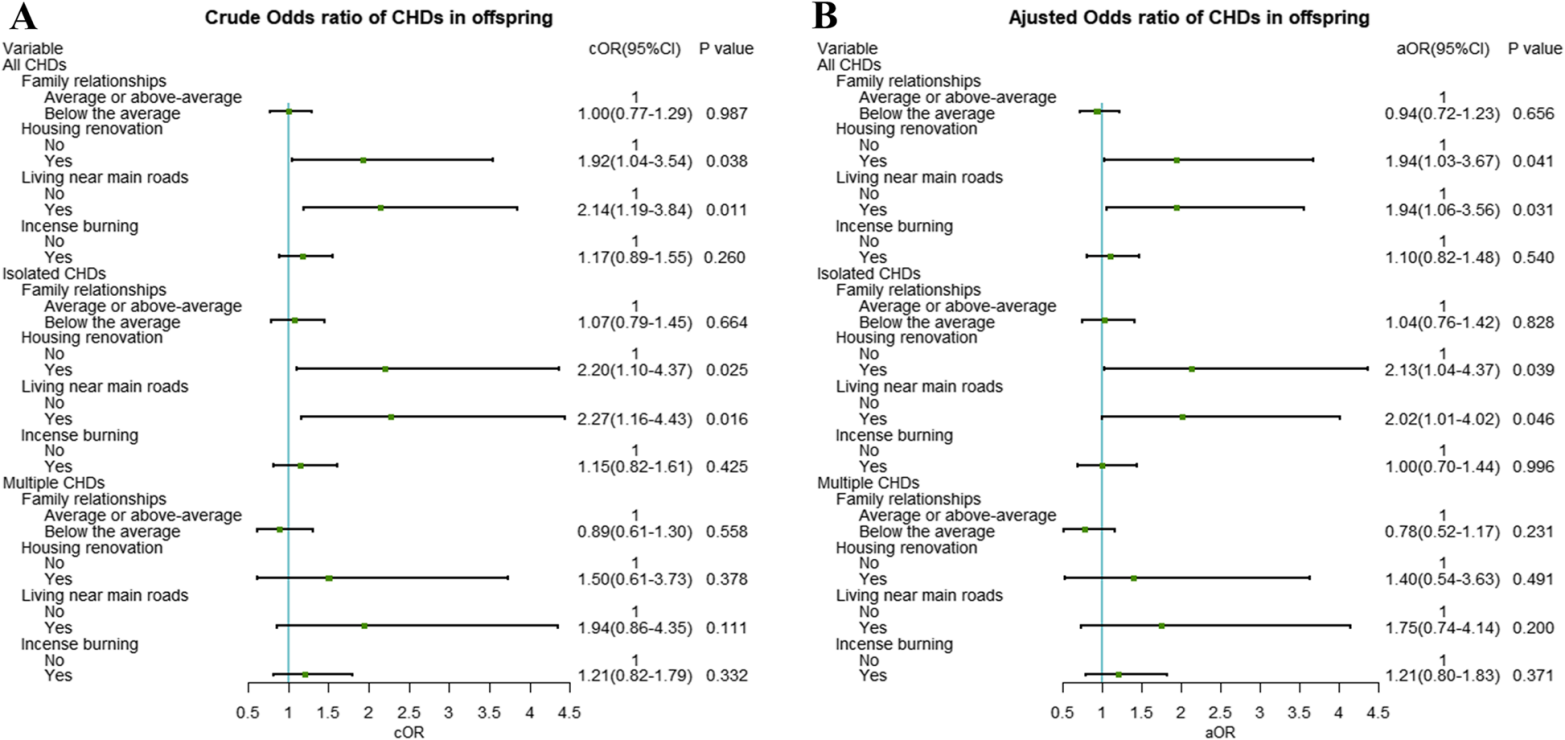
Univariate and Multivariate analysis for maternal environmental exposures associated to all congenital heart defects (CHDs), isolated and multiple CHDs. **A** crude odds ratios and 95% confidence intervals for maternal environmental exposures and all CHDs, isolated and multiple CHDs. **B** adjusted odds ratios and 95% confidence intervals for maternal environmental exposures and all CHDs, isolated and multiple CHDs

Figure 2 shows the associations of maternal environmental exposures during the periconceptional period with offspring CHDs. Without adjusting for other variables, housing renovation (cOR = 1.92, 95%CI = 1.04–3.54) and living near main roads (cOR = 2.14, 95%CI = 1.19–3.84) was positively associated with all CHDs in offspring. Moreover, housing renovation (cOR = 2.20, 95%CI = 1.10–4.37) and living near main roads (cOR = 2.27, 95%CI = 1.16–4.43) were also associated with isolated CHDs in the unadjusted models rather than multiple CHDs.

After adjusting for maternal age at delivery, paternal age at delivery, child sex, family monthly income, gravidity, parity, infant with preterm birth, infant birth weight, maternal passive smoking, maternal folic acid use, mode of conception, threatened abortion, maternal history of internal diseases, maternal history of CHD, maternal reproductive history of birth defects, taking medicine, maternal hyperthyroidism, and maternal reproductive history of birth defects, living near main roads [adjusted OR (aOR), 1.94; 95% CI, 1.06–3.56] and housing renovation (aOR,1.94; 95% CI,1.03–3.67) during the periconceptional period were still associated with higher risk of all CHDs with offspring. In addition, living near main roads (aOR, 2.02; 95% CI, 1.01–4.02) and housing renovation (aOR,2.13; 95% CI,1.04–4.37) during periconceptional period were also associated with isolated CHDs in the multivariate models rather than multiple CHDs.

However, no association was found between family relationships and incense burning and all CHDs in offspring in both the unadjusted models and adjusted models, similar results were also found in isolated and multiple CHDs.

### Association of maternal periconceptional environmental exposure and specific isolated congenital heart diseases

**Fig. 3:**
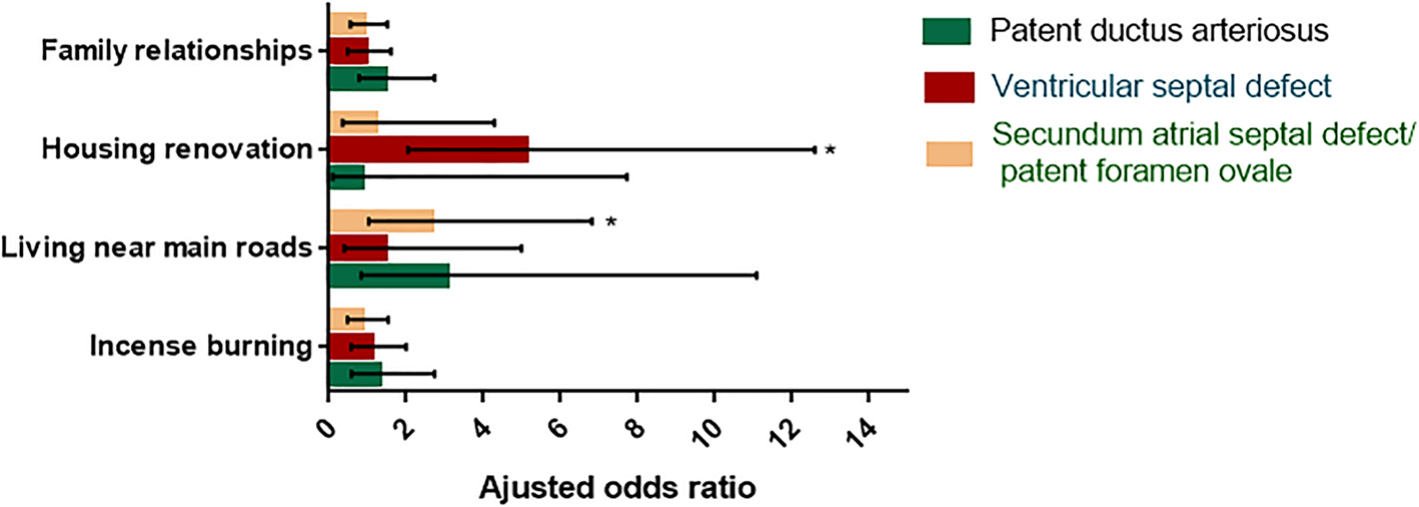
Multivariate analysis for maternal environmental exposures associated to isolated congenital heart defects by different subtypes

We further analyzed the risk factors for isolated CHDs by major subtypes of this study (Fig. 3). Living near main roads was positively associated with secundum ASD/PFO (aOR:2.65, 95%CI = 1.03–6.82) in addition to PDA (aOR:3.05, 95%CI = 0.84–11.08) and VSD (aOR:1.41, 95%CI = 0.40–4.99). Moreover, housing renovation was strongly associated with VSD (aOR:5.08, 95%CI = 2.05–12.60) in addition to PDA (aOR:0.85, 95%CI = 0.09–7.72) and secundum ASD/PFO (aOR:1.23, 95%CI = 0.35–4.29).

However, no significant association was detected between family relationships and incense burning and the risk of these three subtypes of isolated CHDs.

## Discussion

In this study, living near main roads and housing renovation during the periconceptional period were positively associated with all CHDs and isolated CHDs in offspring in addition to multiple CHDs. Additionally, living near main roads was positively associated with secundum ASD/PFO and housing renovation was strongly associated with VSD in further analysis. However, no significant association was found between family relationships and incense burning and all CHDs, similar results were also found in isolated and multiple CHDs as well as three subtypes of CHD.

The univariate analyses found that mothers with higher parity might have lower prevalence of CHDs in offspring. Similarly, Wang et al. [35] found that higher parity groups showed a decreased risk of isolated CHDs. Moreover, we found that mothers who reported having a lack of folic acid use, passive smoking during pregnancy, threatened abortion, maternal history of CHD, take medicine during the first trimester, assisted reproduction, infant with preterm birth have a higher prevalence of CHDs in offspring, these findings were also consistent with previous studies which suggested that mothers with these characteristics had an increased risk of having offspring with CHDs [12, 14, 36–38]. Furthermore, maternal history of internal diseases and hyperthyroidism were positively associated with higher risk for CHDs in offspring. Considering the host of adverse consequences that are linked with CHDs, recognizing women who are at high risk of having CHDs in offspring is critical. Specifically, we found that females with these characteristics as follows, such as lower parity, infants with preterm birth, maternal passive smoking during pregnancy, lack of maternal folic acid use, assisted reproduction, threatened abortion, as well as have a history of internal diseases, CHD, hyperthyroidism, and reproductive history of birth defects before pregnancy may be more prone to have CHDs in offspring. Hence, we suggest that clinicians should pay more attention to the high-risk women with disadvantageous characteristics mentioned above to reduce the potential risk of CHDs in offspring.

Previous studies have showed that living near major roads might have adverse effects on health [39, 40]. However, limited evidence on its relationship with birth defects has been reported. We reported that living near main roads during the periconceptional period was associated with risk of all CHDs, isolated CHDs and secundum ASD/PFO. Gong et al. [41] found that maternal exposure to loud noise also increased the incidence of CHDs. The increased risk for CHD occurrence from maternal exposure to living near main roads may be due to traffic-related air pollution, such as PM2.5, which has been reported to enhance the risk of CHDs in the offspring of exposed mothers [42]. Another possibility is that living near main roads increases exposure to noise, and it has been found that maternal exposure to loud noise also increases the incidence of CHDs [41]. Further studies could explore the relationship between different distances to the nearest main road and the existence of CHDs in offspring.

Furthermore, the data presented in this study also illustrated that housing renovation during periconceptional period was related to a greater risk of all CHDs, isolated CHDs and VSD. Similarly, previous studies also found that maternal exposure to housing renovation was associated with CHDs in offspring [1, 43, 44]. During or after house renovations, volatile organic compounds (VOCs) as well as heavy metals might be emitted from dyes and paints. In addition, formaldehyde and trichloroethylene (TCE) can be released indoors from air boards and plywood [45–47]. Hjortebjerg et al. [48] suggested that maternal nonoccupational exposure to paint fumes might be linked with congenital abnormalities. Furthermore, experimental studies have demonstrated that TCE can result in the developmental abnormalities in the hearts of avian embryos and mouse embryos. The mechanism by which housing renovation increases the risk of CHDs is not fully understood. The above evidence may partially account for the mechanism of CHDs caused by house renovation at least, and the biological plausibility and specific toxicological mechanism of different environmental contaminants of housing renovation should be further confirmed.

### Limitations

Several limitations in this study should be mentioned. First, to generate more “homogeneity” due to combining all the CHDs together in this study, specific CHD subtypes such as isolated PDA, VSD, secundum ASD/PFO were measured for further association analysis. However, the statistical power for specific CHD subtypes was limited due to the relatively small sample size. Second, due to the use of a case–control study method to assess maternal periconceptional environmental exposure history, recall bias could not be ruled out. Third, family relationship was measured as a binary classification variable due to the few numbers in the poor and very poor category. Therefore, further studies could extend the sample size to explore the association between poor family relationships and CHD in offspring. Finally, the frequency of burning incense was not investigated in this study; therefore, the dose–response effect of maternal burning incense on offspring with CHDs could be further assessed.

### Strengths

Despite these limitations, our study has several strengths. First, to our knowledge, the current research is the first to explore the role of maternal exposure to family relationships and incense burning and in the risk of CHDs in offspring. Furthermore, the participating women were studied from the first 3 months before pregnancy to the first trimester of pregnancy in our research, which expands and clarifies the susceptibility time of maternal environmental exposure. Additionally, given that maternal periconceptional environmental exposure is a modifiable risk factor, this study might be useful in guiding screening and intervention strategies for women who are at high risk of having CHDs in offspring. We suggest that women who are pregnant or planning pregnancy should avoid maternal exposure to living near main roads and housing renovation.

## Conclusions

Living near main roads and housing renovation during the periconceptional period were associated with a higher risk of congenital heart disease in offspring. Additionally, living near main roads was positively associated with secundum ASD/PFO and housing renovation was strongly and positively associated with VSD. Considering the limited sample size, further study is needed to extend the sample size to explore the effects of time and frequency of burning incense and family relationships on CHDs in offspring.

## Supplementary Information

**Additional file 1:** **Supplementary Table 1.** The details of types of 675 CHD cases.

## Data Availability

The datasets used and/or analyzed during the current study are available from the corresponding author upon reasonable request.

## Abbreviations

ASD: Atrial septal defect
CHD: Congenital heart defects
Cis: Confidence intervals
HPA: Hypothalamo-pituitary-adrenal
ICD-10-CM: 10th Revision, Clinical Modification
ORs: Odds ratios
PDA: Patent ductus arteriosus
PFO: Patent foramen ovale
PM2.5: Fine particulate matter
TCE: Trichloroethylene
VOCs: Volatile organic compounds
VSD: Ventricular septal defect
