# Supplementary material for: Maternal periconceptional environmental exposure and offspring with congenital heart disease: a case–control study in Guangzhou, China
Source: BMC Pregnancy Childbirth. 2023 Jan 24;23:57. doi: 10.1186/s12884-023-05355-5 (PMC9872400; doi:10.1186/s12884-023-05355-5)
Supplement: Supplementary file 1 — Additional file 1: Supplementary Table 1. The details of types of 675 CHD cases. [file 12884_2023_5355_MOESM1_ESM.docx]

| **Supplementary Table 1 The details of types of 675 CHD cases.** | |
| --- | --- |
| **Types of CHDs** | **Number (%)** |
| **All CHDs** | 675(100) |
| **Isolated CHDs** | 405(60.0) |
| **Congenital abnormalities of the great arteries** |  |
| Patent ductus arteriosus(PDA) | 68(10.1) |
| Aortic coarctation | 3(0.4) |
| Pulmonary valve stenosis | 1(0.1) |
| Other congenital malformations of the great arteries | 15(2.2) |
| **Congenital septal malformations** |  |
| Atrioventricular septal defects (AVSD) | 5(0.7) |
| Ventricular septal defect (VSD) | 114(16.9) |
| Secundum atrial septal defect(ASD)/patent foramen ovales(PFO) | 147(21.8) |
| Other congenital cardiac septal malformations | 13(1.9) |
| **Other CHDs (eg. persistent left superior vena cava, congenital stenosis of aortic valve, congenital pulmonary valve stenosis, etc)** | 39(5.8) |
| **Multiple CHDs** | 270(40.0) |
